# Functional Study of One Nucleotide Mutation in Pri-MiR-125a Coding Region which Related to Recurrent Pregnancy Loss

**DOI:** 10.1371/journal.pone.0114781

**Published:** 2014-12-05

**Authors:** Yi Hu, Zheng-Hao Huo, Chun-Mei Liu, Shi-Guo Liu, Ning Zhang, Kun-Lun Yin, Lu Qi, Xu Ma, Hong-Fei Xia

**Affiliations:** 1 Reproductive and Genetic Center of National Research Institute for Family Planning, Beijing, China; 2 Graduate School; Peking Union Medical College, Beijing, China; 3 Chinese Academy of Sciences Key Laboratory of Pathogenic Microbiology and Immunology, Institute of Microbiology, Chinese Academy of Sciences, Beijing, China; 4 Department of Biotechnology, School of Basic Medical Science, Ningxia Medical University, Yinchuan, Ningxia, China; 5 Affiliated hospital of medical college of Qing Dao university, Qingdao, China; University of Texas, MD Anderson Cancer Center, United States of America

## Abstract

MicroRNAs (miRNAs) are short non-coding RNAs which modulate gene expression by binding to complementary segments present in the 3′UTR of the mRNAs of protein coding genes. MiRNAs play very important roles in maintaining normal human body physiology conditions, meanwhile, abnormal miRNA expressions have been found related to many human diseases spanning from psychiatric disorders to malignant cancers. Recently, emerging reports have indicated that disturbed miRNAs expression contributed to the pathogenesis of recurrent pregnancy loss (RPL). In this study, we identified a new mutation site (+29A>G, position relative to pre-miR-125a) by scanning pri-miR-125a coding region in 389 Chinese Han RPL patients. This site was co-existed with two polymorphisms (rs12976445 and rs41275794) in patients heterogeneously and changed the predicted secondary structures of pri-miR-125a. Subsequent *in vitro* analysis indicated that the A>G mutation reduced mature miR-125a expression, and further led to less efficient inhibition of verified target genes. Functional analysis showed that mutant pri-mir-125a can enhance endometrial stromal cells (ESCs) invasive capacity and increase the sensitivity of ESCs cells to mifepristone. Moreover, we further analyzed the possible molecular mechanism by RIP-chip assay and found that mutant pri-mir-125a disturbed the expression of miR-125a targetome, the functions of which includes embryonic development, cell proliferation, migration and invasion. These data suggest that A>G mutation in pri-miR-125a coding region contributes to the genetic predisposition to RPL by disordering the production of miR-125a, which consequently meddled in gene regulatory network between mir-125a and mRNA.

## Introduction

Human reproduction is remarkably inefficient compared with other mammalian species and approximately 70% of spontaneous conceptions are lost prior to completion of the first trimester [1]. Half of the cases of spontaneous abortion are associated with fetal chromosomal abnormalities, endocrine disorders, immune factors, uterine structural abnormalities, infections, chemical agents and psychological factors. However, the etiology of approximately 50% of spontaneous abortion is not well known. As a common and distressing disorder, recurrent pregnancy loss (RPL) affects about 1–5% of the reproductive age couples worldwide [2]. Despite the failure in clarifying the major causes occurred in up to 50% of cases [3], genetic influence is commonly thought to be one of the important factors leading to RPL.

MicroRNAs (miRNAs) are small noncoding RNA molecules that function as negative regulators of the expression of protein coding genes. Following transcription by RNA polymerase II, primary miRNA (pri-miRNA) are processed by Drosha and converted into a ∼70 nt hairpin precursor miRNA (pre-miRNA), in mammalian cells [4]. Through the interaction with exportin-5 and Ran-GTP, the pre-miRNA is transported into the cytoplasm and where they are further processed by another RNase III, Dicer and finally turn into mature miRNA of ∼22 nt. Mature miRNAs can be loaded onto the RNA-induced silencing complex (RISC) which is able to recruit target mRNA and then initiate consequent translation inhibition or mRNA degradation. Argonaute (AGO) proteins, which bind directly to mature miRNAs, are a central and catalytic component of RISC. Recently, anti-AGO ribonucleoprotein immunoprecipitation-microarray profiling (RIP-Chip) were used to study the global patterns of mRNAs recruitment to RISC [5], [6].

Increasing evidence shows that single nucleotide polymorphisms (SNPs) or mutations in miRNA coding region may alter miRNA expression and/or maturation and be involved in the occurrence of diseases [7], [8]. Our previous work indicated that two SNPs in (rs12976445, rs41275794) pri-miR-125a are associated with human RPL. In this study, we confirmed a mutation site in 6 RPL patients which co-existed with the rare allele of these two SNP sites and caused a more significant reduction of miR-125a expression. Functional analysis showed that pri-mir-125a mutant genotype can enhance endometrial stromal cells invasive capacity and increase the sensitivity of cells to mifepristone-induced the inhibition of cell proliferation compared with normal genotype. These data suggest that A>G mutation in pri-miR-125a coding region co-existed with the rare alleles of rs41275794 and rs12976445 contributes to the genetic predisposition to RPL by disordering the production of miR-125a, which consequently meddled in gene regulatory network between mir-125a and mRNA.

## Results

### Sequencing and validation discovery

**Figure 1:**
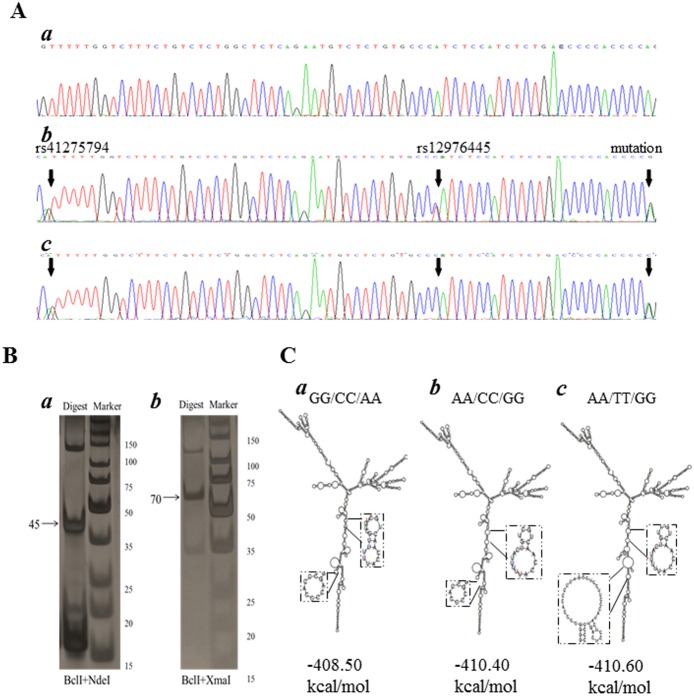
Detect mutation site in pri-miR-125a and identify the hyplotypes. (A) An example of chromatographs showing the heterozygosis on rs41275794 and rs12976445 and the mutation site in pri-miR-125a. Black arrows indicate heterozygous site. (B) Haploytpe analysis by PCR-RFLP. Black arrows indicate double digest products. (C) Secondary structure prediction. The mutation site and two SNPs (rs41275794 and rs12976445) cause two apparent changes in loop size and a lower predicted ΔG from −408.50 kcal/mol to −410.60 kcal/mol. The mutation site with rs41275794 also causes changes in loop size and a lower predicted ΔG from −408.50 kcal/mol to −410.40 kcal/mol.

In the previous study, we found one mutation site in pri-miR-125a in two RPL patients in a Chinese-Han population of Beijing (North China). To further validate our discovery, we collected another population consist of 172 patients and 221 controls from Ningxia (Northwest China) and scanned the same region of pri-miR-125a. The genotype distributions of four variants in pri-miR-125a in the complete association samples comprising 389 RPL patients and 652 ethnically matched control individuals were shown in Table 1. The mutation site (Chr19∶52196480, A>G) only existed in 6 patients with heterogeneous pattern and not found in controls and 1000 Genomes data base. As shown in Fig. 1A, the mutation site co-exists with 2 SNPs (rs41275794 and rs12976445) in 5 patients (2 patients from Beijing and 3 patient from Ningxia) and one SNP (rs41275794) in another patient (from Ningxia) as heterogeneous pattern.

**Table 1:** Variants in pri-miR-125a and their frequencies.

Gene	Chro.location^a^	Alleles^b^	dbSNP	Minor allelefrequency			Genotypedistribution		
				Con%	RPL%	p	Con	RPL	p
miR-125a	Chr.19∶52196210	G>A		0.71	0.95	0.57	0/9/622	0/7/363	0.85
	Chr19∶52196453	C>T	rs12976445	19.9	28.9	<0.01	12/227/392	2/210/158	<0.01
	Chr19∶52196409	G>A	rs41275794	15.06	32.3	<0.01	9/172/450	10/219/141	<0.01
	Chr19∶52196480	A>G		0.00	0.81	<0.01	0/0/631	0/6/364	0.015

Abbreviations: con, control individuals; RPL, recurrent pregnancy loss patients.

a Chromosomal positions from NCBI build 37.

b Major allele > minor allele on the + strand.

Variants and genotypes that are uniquely present in patients or control individuals are underlined.

### Haplotype identification

To identify the haplotype of the mutation and two SNPs, we used twice double restriction endonucleases cut analysis (Fig. 1B). Separating by polyacrylamide gel electrophoresis, we can found double digested products in these two PCR products. That means the mutation site existed with the two SNPs in one chromosome. So the haplotype of mutated DNA chain are A-T-G (H2) and A-C-G (H3).

### The A>G Mutation changes the predicted pri-miR-125a secondary structures and enhances their molecular stability

The secondary structure of 1016 bp pri-miR-125a sequence was predicted using RNAfold web server (http://rna.tbi.univie.ac.at/cgi-bin/RNAfold.cgi). The mutation site in H2 genotype pri-miR-125a cause two apparent changes in loop size and a lower predicted ΔG from −408.50 kcal/mol to −410.60 kcal/mol. The site mutation in H3 also causes changes in loop size and a lower predicted ΔG from −408.50 kcal/mol to −410.40 kcal/mol (Fig. 1C).

### The A>G change decreases miR-125a expression in transfected cells

**Figure 2:**
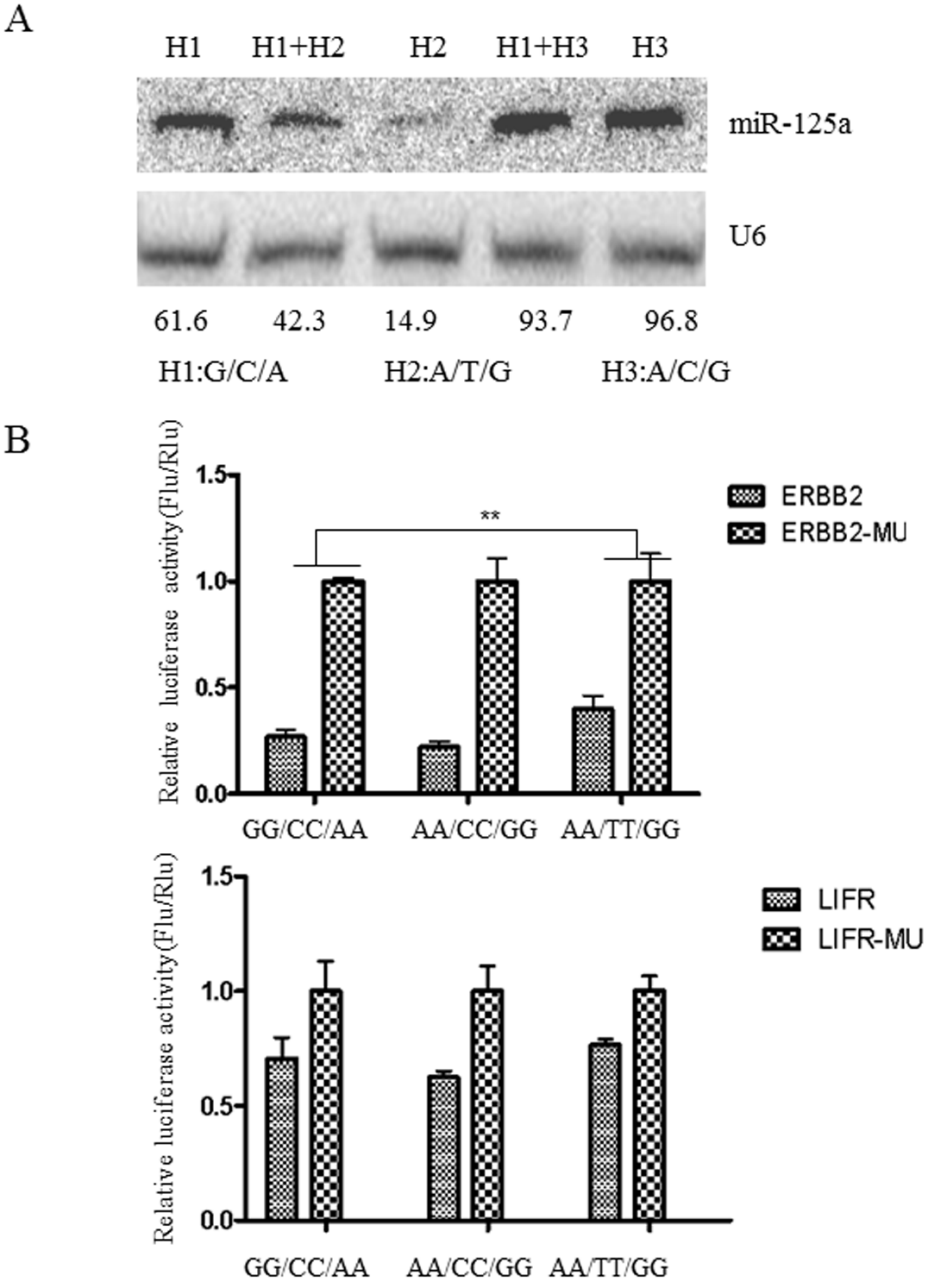
The mutation site reduced the expression of miR-125a and up-regulate the expression of miR-125a target genes. (A)The result of northern blot indicates that mutation site co-exist with two SNPs reduce miR-125a expresson. (B) The 3′UTR of ERBB2 and LIFR were cloned into downstream of the firefly luciferase gene in pGL3-Control Vector. Different genotype miR-125a expression vector were transfected into HEK293T cells with pRL-TK and pGL3 vector which contained 3′UTR sequence of ERBB2 or LIFR. Luciferases activities were detected 24 h after transfection.

To study the function of the mutation site, three haplotypes pri-miR-125a expression plasmids were constructed and transfected into HEK293T cells. Northern blot was used to detect the expression of mature miR-125a. As shown in Fig. 2A, the expression of mature miR-125a was different among these three genotypes. MiR-125a expression level of G-C-A haplotype (the most exist in Chinese women) haplotype is nearly 4 fold higher than A-T-G. Mature miR-125a expressed from A-C-G is nearly 1.6 fold higher than G-C-A haplotype.

### Impact of nucleotides variants on miR-125a target genes

To investigate the functional consequences of miR-125a A>G mutation on its target genes, we chose two confirmed miR-125a target genes. One was ERBB2, proved by Scott’s group [9] and another was LIFR, confirmed by our study[10]. The 618 nt 3′-UTR of ERBB2 and a 1043 nt 3′-UTR of LIFR, which contain miR-125a target sites, were PCR-amplified from human genomic DNA and cloned into the downstream of the stop codon of firefly luciferase gene. Deleting putative miR-125a binding region in the 3′UTR of ERBB2 (ERBB2-Del) or LIFR (LIFR-Del) was used as corresponding control. These reporter constructs were transiently transfected into HEK293T cells together with an expression plasmid containing alone genotype miR-125a. The results were analyzed by using multiple comparison/post-hoc tests in ANOVA. Levene's test is used to assess variance homogeneity, which is a precondition for parametric tests such as the t-test and ANOVA.

The results analyzed by Levene’s Test showed that the variances are homogeneous in LIFR (P = 0.322) and ERBB2 (P = 0.136). From the results in Fig. 2B, we found that the activity of firefly luciferase was significantly increased (P = 0.003) in cells co-transfected with ERBB2 and pri-miR-125a A-T-G haplotype when compared with pri-miR-125a G-C-A haplotype. The firefly activity was a little reduced by the pri-miR-125a A-C-G haplotype, but the difference was not significant (P = 0.36). When cells were co-transfected with LIFR and different pri-miR-125a genotypes, the activity of firefly luciferase was not significantly changed in any one group (LIFR and G-C-A, A-C-G or A-T-G genotype; Fig. 2B). The results indicated that the A>G mutation with two SNPs can affect the expression of ERBB2 by changing the production of miR-125a. ERBB2 is necessary for induction of carcinoma cell invasion 14, so we speculated that pri-miR-125a mutant genotype may enhance cell invasive capacity via increasing the expression of ERBB2.

### Mutant pri-miR-125a enhances endometrial stromal cells (hESCs) invasive capacity

**Figure 3:**
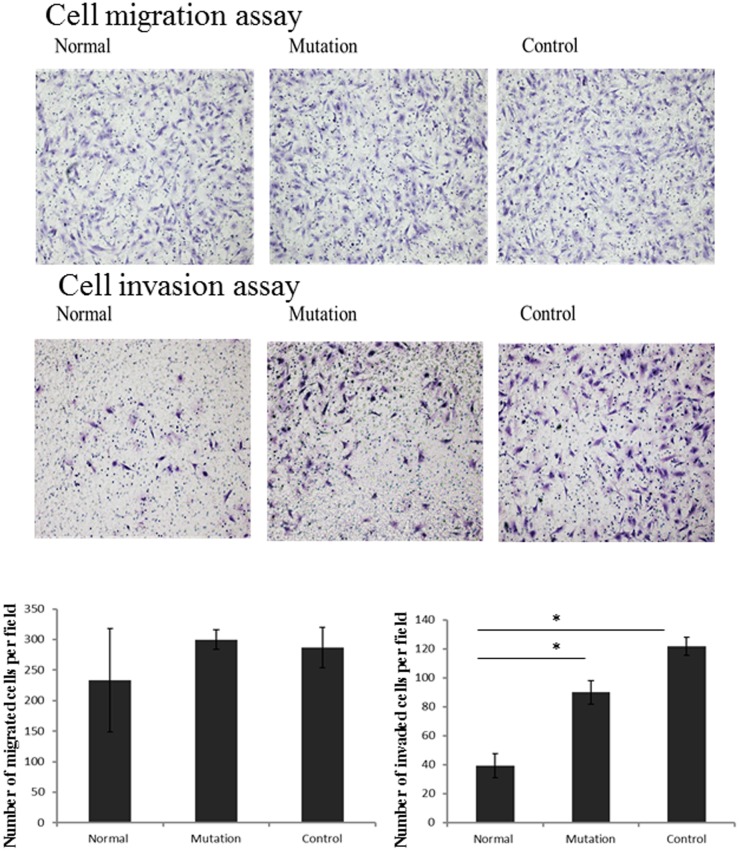
Cell migration and invasion assay. For cell migration assay, hESC cells were seeded to the top chamber, medium with high concentration of serum was added to the bottom. 12 h later, cells on the lower surface were stained with hematoxylin-eosin. Ten random fields were selected to determine the average number of cells per view field. For cell invasion assays, the procedure was similar to the cell migration assay, except transwell membranes were precoated with Matrigel.

Trophoblast invasion into the decidual stroma and spiral arteries is one of the crucial steps in human embryo implantation and establishment of pregnancy. In order to validate the effect of the mutation on cell migratory and invasive behavior, a typical transwell assay was employed (Fig. 3). The results indicated that there was no significant difference of the effect on hESCs migratory capacity between normal and mutant genotypes pri-miR-125a. However, the invasive ability of hESCs transfected with pri-miR-125a normal genotype was significantly lower than that transfected with pri-miR-125a mutant genotype (P<0.01), revealing that pri-miR-125a mutant genotype, i.e. down-regulation of miR-125a, can promote cell invasion via up-regulation of miR-125a target genes.

### Mutant pri-miR-125a increases the sensitivity of hESCs to mifepristone

**Figure 4:**
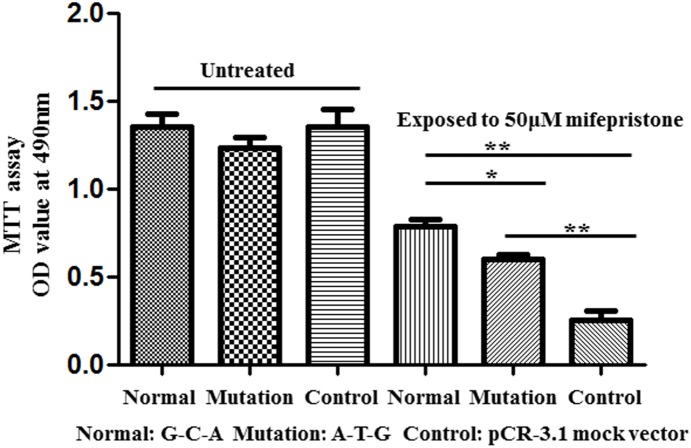
Cell proliferation assay. hESCs were seeded in 96-wells plate and then transfected with different genotypes miR-125a expression vector. 24 h after transfection 50 µM mifepristone was added into the wells in mifepristone exposed groups. And twenty microliters MTT (5 mg/ml) were added to each well 36 h after transfection and cells were incubated for further 4 h. The absorbance was recorded at A570 nm with a 96-well plate reader after the DMSO addition.

During pregnancy, cell proliferation is common events in the maternal-fetal interface. To understand the effect of mutant pri-miR-125a on cell growth, MTT assay was used. As shown in Fig. 4, the proliferation of hESCs was not visibly changed when cells were transfected with normal, mutation pri-miR-125a expression vectors or pCR3.1 plasmid.

Mifepristone is a synthetic steroid compound used as a pharmaceutical which is a progesterone receptor antagonist used as an abortifacient in the first months of pregnancy [11]. There are evidence that mifepristone treatment can inhibit endometrial proliferation in vivo [12] and Ishikawa endometrial adenocarcinoma cells proliferation in vitro [13]. Therefore, we treated hESCs with mifepristone to simulate abortion status and further research the effect of pri-miR-125a mutant genotype on cell growth (Fig. 4). When exposed to 50 µM mifepristone, cell proliferation of all the groups were significantly inhibited compared with untreated groups. Meanwhile, cells transfected with pri-miR-125a normal (P<0.01) and mutant genotype (P<0.01) had a higher viability than that transfected with pCR3.1 empty vector, implying that the up-regulation of miR-125a can attenuate mifepristone-mediated the suppression of cell growth. However, the cells transfected with pri-miR-125a mutant genotype had lower proliferation capacity than that transfected with pri-miR-125a normal genotype (P<0.05), displaying that the sensitivity of cells to mifepristone-induced the inhibition of cell proliferation was increased by mutant pri-miR-125a.

### RIP-chip assay to detect the effect of different genotype pri-miR-125a on gene expression profile in cells

In order to further explore the possible molecular mechanism that pri-miR-125a mutant genotype induced RPL, RIP-chip assay was used to detect the effect of different pri-miR-125a genotypes on target mRNAs expression profiles on a genome-wide scale.

**Figure 5:**
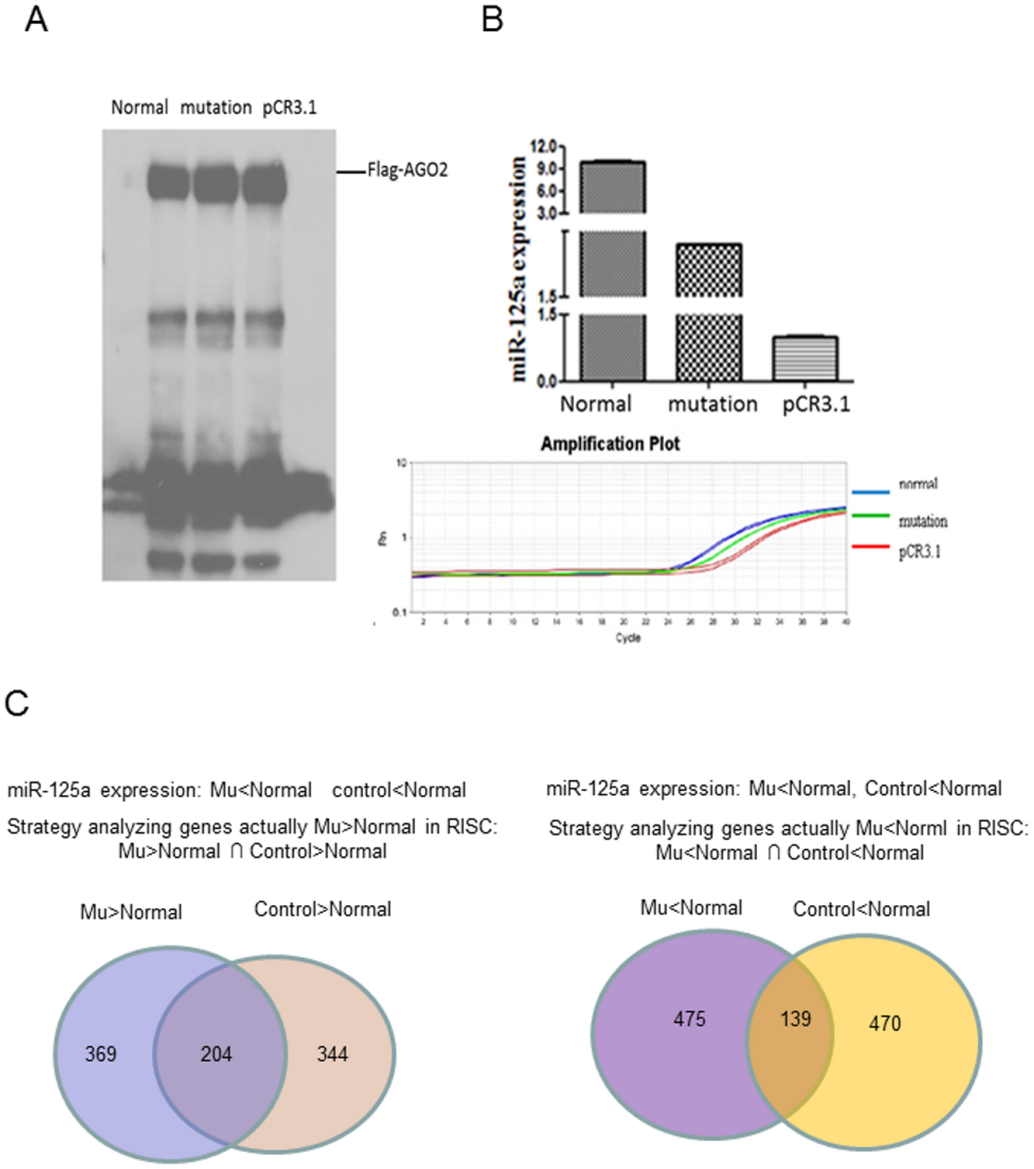
RIP-chip analysis to detect the altered repression profile enriched in RISC complex. (A) Western blot analysis to detect Flag-AGO2 quantity in the precipitate. Real time PCR used to detect miR-125a quantity in the precipitate. (B) The analyze strategies of the repression profile.

Western blots from co-IPs (Fig. 5A) showed that Flag-AGO2 was isolated with the anti-Flag antibody and the quantity of Flag-AGO2 was no significant different among these 3 groups. The amount of miR-125a in precipitation was tested by using real-time PCR. As shown in Fig. 5B, the quantity of miR-125a in normal genotype and mutant genotype are 10.3 fold and 2.8 fold change compared with mock vector, respectively. The mRNA was eluted from RISC in Flag-AGO2 stable expression cell line co-transfected with A-T-G or G-C-A genotype or mock vector. The expression pattern of mRNAs isolated from co-IP was analyzed by using Gene Expression Profiling Human OneArray HOA 5.1 chip (Phalanx Biotech). In order to eliminate the background noise of genes expression, we adopted the strategy as follows (Fig. 5C): (1) genes more enriched in mutation group (because of less mir-125a expression) compared with normal genotype should be removed false positive results by intersecting with genes more enriched in mock vector group compared with normal. (2) Genes more diminished in mutation group compared with normal should be intersected with genes more enriched in normal genotype group compared with mock vector. Two-sample Student’s t-test was used to analyze differentially expressed genes between pri-miR-125a normal and mutant genotypes, and P value <0.05 was considered significant. Results showed that 204 genes were enriched and 139 genes were diminished in mutation group (Table S1 and Table S2).

### GO analysis indicated 9 genes directly related to embryo development were enriched in mutation group

**Figure 6:**
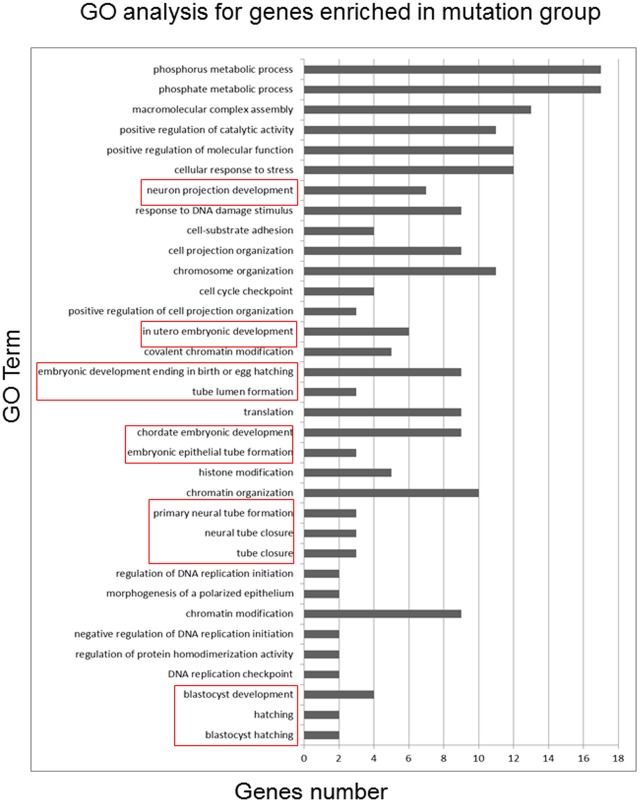
GO analysis of the genes enriched in RISC in mutant genotype group. Genes enriched in RISC in mutant genotype were analyzed use David online tools. The GO terms were listed on the left from down to up according P value from small to large.

To assign biological meaning to the group of genes with changed enrichment, the subset of genes which met the above criteria were analyzed with the Gene Ontology (GO) classification system, using DAVID software (http://apps1.niaid.nih.gov/david/) [14]. Overrepresentation of genes with altered expression within specific GO categories was determined using the Fisher exact test (P<0.05). After GO analysis we found 9 embryo development related genes (PTK7, CHD8, GRN, TAB1, NDEL1, RBBP8, PSMC4, TSC2, and VANGL2) enriched in RISC complex in the mutation group, which means the expression of those genes should be more seriously repressed than the normal genotype cells(Table S1). As shown in Fig. 6, highlighted by red box, their functions involve blastocyst hatching, blastocyst development, neural tube closure, in utero embryonic development and so on.

**Figure 7:**
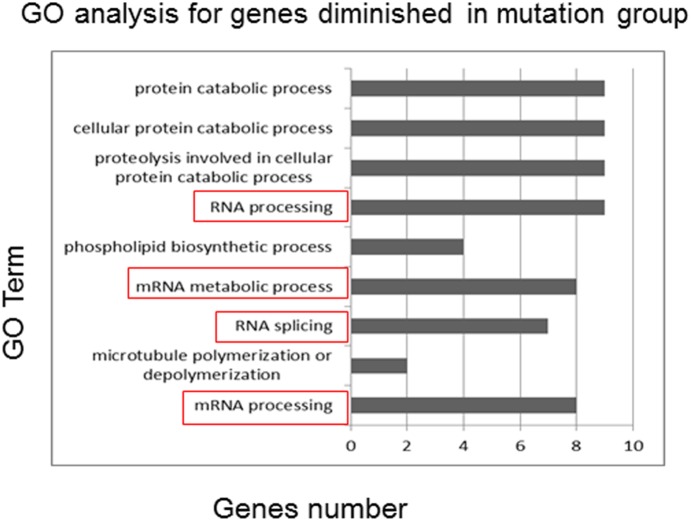
GO analysis of the genes diminished in RISC in mutant genotype group. Genes diminished in RISC in mutant genotype were analyzed use David online tools. The GO terms were listed on the left from down to up according P value from small to large.

After GO analysis we found some RNA processing, splicing and metabolism associated genes (SCAF1, SSU72, U2AF1, U2AF2, ADAD2, NONO, SNRPB2, SF3B14 and SKIV2L2) were more diminished in RISC complex in the mutation group (Fig. 7 and Table S2), which means the expression of those genes should be promoted more than the normal genotype cells. Among them, U2AF1, U2AF2 and SNRPB2 are components of spliceosome. Although there no direct evidence of the relationship between spliceosome and RPL, the function of spliceosome is broad and associates with serious diseases [15].

## Discussion

In this study, we provide the first piece of evidence that link a new nucleotide mutation of pri-miR-125a to human RPL susceptibility. This new mutation site co-exist with the rare alleles of two SNPs, reduces the expression of miR-125a fiercely and alleviates the expression repression of miR-125a target genes.

ERBB2 is a member of the epidermal growth factor receptor (EGFR/ErbB) family, and usually over expressed in many kind of cancer cells and promoting cell proliferation. According to the function of ERBB2, the reduction of miR-125a expression should promote cell proliferation activity. But in contrary, our functional study using hESCs get the opposite results. When exposed in mifepristone, pri-miR-125a mutant genotype has a negative influence on cell proliferation ability. So we formulate a hypothesis that there exist some other important mechanisms about the function of miR-125a reduction.

Since the function of miRNA mostly depend on repressing the expression of its target genes and one miRNA always have hundreds of targets, so we want to get a clue by using RIP-Chip to identify the changes of “repression profile” in RISC complex in mutant pri-miR-125a genotype group. The result of RIP-chip assay shows that many cell proliferation promoting factors are recruited in RISC of mutant group and in the same time, some suppressors of cell proliferation are diminished. So that can explain the negative influence by pri-miR-125a mutant genotype on cellular proliferation mechanisms is independent of ERBB2.

In our human beings, the incidence of embryo wastage and pregnancy loss is extraordinarily high [15], [16]. From a clinical perspective, miscarriage, whether sporadic or recurrent, is widely viewed as a dichotomous disorder, attributed either to chromosomal or other developmental abnormalities in the embryo or to uterine factors. By gene scanning and functional study, we find a site mutation in pri-miR-125a which cause 204 genes enriched and 139 genes diminished in RISC complex. 9 of them are embryo development essential genes and disturbed expression of them can indicate embryo death or fetal deformity. So here, we suggest that, the reduced expression of miR-125a may induce RPL in mainly three ways: (1) the reduced miR-125a may disturb genes expression in maternal endometrium and misguide the mother to reject normal embryos. (2) Chromosome contain mutant miR-125a can be inherited into the egg, and disturb the embryo development. (3) There are evidences that miRNAs can be secreted into extracellular matrix [17]–[19]. The embryo development may also be affected by secretary reduced mother miR-125a.

In conclusion, we found one nucleotide mutation co-existed with two SNPs reduced the expression of miR-125a, disturb the expression of miR-125a target genes and enhanced cell invasion ability. To our knowledge, this is the first report about site mutation in pri-miRNA which is associated with human RPL. Although the precise roles of this site mutation require further experimental exploration, this discovery may give new sight into understanding of unexplained RPL development and create an opportunity to approach the diagnosis and treatment of unexplained RPL.

## Materials and Methods

### Patients and control samples

This study contains 389 Han-Chinese patients with two or more consecutive spontaneous abortions and 652 ethnically matched controls. 217 patients and 431 healthy controls were recruited from the Peking Union Medical College, China. 182 patients and 221 healthy controls were recruited from the Ningxia Medical University, China. The patients and controls are all Han-Chinese population. We exclude many factors which are likely to cause this medical condition by blood test, including virus infection (TORCH) test, immune antibodies (ANA/ACLA) test, glucose level, T3/T4/TSH and sexual hormones level in maternal blood. Also we check the couple's chromosomal karyotype and blood type. Meanwhile perform the ultrasound scan for the patient to confirm no anomaly of reproductive system existed. In addition, we carry out the routine semen analysis to exclude the factor likely coming from the patients' partner. Finally we exclude out the patients who have genetic defects or hereditary diseases in their family history. Controls were individuals of proven fertility, with normal menstrual cycles and ovary morphology, without the history of subfertility treatment. DNA was extracted from the blood using TIANamp Blood DNA Kit (TIANGEN, Beijing, China). This study was approved by the Ethics Committee of Research Institute for Family Planning (No. 2011–10) and written informed consents were obtained from all participants.

### Sequencing

DNA specimens were amplified by using standard PCR protocols. The PCR products were sequenced in forward direction with the ABI 3730xl sequencing platform. The sequencing results were analyzed by using DNAMAN and Chromas Lite software. Haploytpe analysis by PCR-RFLP.

The restriction digestion of 112 bp and 119 bp PCR product with BclI and NdeI or with BclI and XmaI enzymes revealed the haplotypes G-C-A, A-T-G, and A-C-G. There are three mutation sites and two mutation sites have to be added respectively in the primers.

### MiR-125a expression vectors and cell transfection

To construct pri-miR-125a expression vectors, fragments (1,016 nt). Corresponding to pre-miR-125a and its flanking regions (previously determined to have the four genotypes) were amplified from human genomic DNA and cloned into the pCR3.1 vector (Invitrogen, Carlsbad, CA, USA). The sequences of four vectors were confirmed by direct sequencing; the only difference was in the SNP sites. The pCR3.1-based plasmid, miR-125a mimic and miR-125a inhibitor were transfected by using lipofectamine 2000 (Invitrogen, Carlsbad, CA, USA) followed the manufacture’s instruction.

### Northern blot analysis

Total RNA was isolated from cultured HEK193T cells with TRIzol reagent (Invitrogen, Carlsbad, CA, USA). 50 µg of total RNA per sample was subjected to electrophoresis on a precast 15% denaturing TBE-urea polyacrylamide gel. And then the RNA was electrophoretically transferred to nylon membranes (Hybond N+; Amersham Pharmacia Biotech, St Albans, Herts, UK). After being UV-cross-linked and baked at 80°C for 30 min, the membrane was prehybridized at 42°C for 4 h and then hybridized with 32P-labeled miR-125a or U6 probes at 40°C overnight. Membranes were washed and exposed to PhosphorImager screens (GE Healthcare Bio-Sciences Corp., Piscataway, NJ, USA). The bands were analyzed using Quantity One software (Bio-Rad, Hercules, CA, USA). All experiments were repeated at least three times.

### 3′-UTR Luciferase Reporter Assays

To generate 3′-UTR luciferase reporter, partial sequence of the 3′-UTR from LIFR and ERBB2 were cloned into the downstream of the firefly luciferase gene in pGL3-Control Vector (Promega, Madison, WI, USA). Deleting miR-125a target sites in the 3′-UTR of LIFR or ERBB2 was used as corresponding control. MiR-125a mimic and miR-125a inhibitor were synthesized by GenePharma Co., Ltd (Shanghai, China). PRL-TK containing Renilla luciferase was co-transfected for data normalization. For luciferase reporter assays, HEK293T cells were seeded in 48-well plates and 24 h later transfected by using lipofectamine 2000 (Invitrogen, Carlsbad, CA, USA). Two days later, cells were harvested and assayed with the Dual-Luciferase Assay (Promega, Madison, WI, USA). Each treatment was performed in triplicate in three independent experiments. The results were expressed as relative luciferase activity (Firefly LUC/Renilla LUC).

### RIP-chip analysis

RIP-chip assay was used to identify the disturbed targets of miR-125a when the site mutation existed. The steps described in supplemental materials and methods.

### Cell migration and invasion assay

A typical Transwell assay (Costar, 6.5 mm diameter, 8 µm pore size) was used. 3×104 cells in 200 µL serum-free medium were seeded to the top chamber and 500 µL medium with high concentration of serum was added to the bottom. After 12 h, Filters were then submerged in 4% PFA for 15 min and cells on the upper surface were removed by cotton swabs. The cells on the lower surface were stained with hematoxylin-eosin. Ten random fields were selected to determine the average number of cells per view field.

For cell invasion assays, the procedure was similar to the cell migration assay, except transwell membranes were precoated with 24 µg/µl Matrigel (BD bioscience, Franklin Lakes, NJ, USA) and the cells were incubated for 8 hr at 37°C in a 5% CO_2_ atmosphere. Cells adhering to the lower surface were counted the same way as the cell migration assay.

### Cell proliferation assay

Cell proliferation was estimated by the MTT assay and conformed by EdU proliferation assay.

### Microarray analysis

Microarray analyses of RNAs isolated from co-IP were performed using Gene Expression Profiling Human OneArray HOA 5.1 chip (Phalanx Biotech). Standard selection criteria to identify differentially expressed genes are established at log_2_ |Fold change| ≥1 and P<0.05. Microarray data are available in the ArrayExpress database (www.ebi.ac.uk/arrayexpress) under accession number: E-MTAB-2394.

### GO analysis

To assign biological meaning to the group of genes with changed enrichment, the subset of genes which met the above criteria were analyzed with the Gene Ontology (GO) classification system, using DAVID software (http://apps1.niaid.nih.gov/david/) [14]. Overrepresentation of genes with altered expression within specific GO categories was determined using the Fisher exact test.

### Statistical analysis

SHEsis11 software was used to calculate genotype deviation from Hardy-Weinberg equilibrium (HWE), and to compare individual allele and genotype frequencies between patients and controls, by a standard χ2 test for independence.

The data of cell proliferation assay, cell invasion assay and real-time PCR were analyzed using SPSS Statistical Package version 16, by One-Way ANOVA test. For the luciferase reporter assays, multiple comparison/post-hoc tests in ANOVA were used.

## Supporting Information

Table S1 Genes enriched in mutant group. This table includes 199 genes enriched in the mutant pri-miR-125a group. Embryo development related genes were taped using red letters. Cell proliferation regulation genes were marked with blue.
(DOCX)

Table S2 Genes diminished in mutant group. This table includes 141 genes diminished in mutant group. RNA processing associated genes are showed in red letters, and cell proliferation, migration and invasion genes used blue.
(DOCX)
