# Supplementary material for: Functional Study of One Nucleotide Mutation in Pri-MiR-125a Coding Region which Related to Recurrent Pregnancy Loss
Source: PLoS One. 2014 Dec 5;9(12):e114781. doi: 10.1371/journal.pone.0114781 (PMC4257728; doi:10.1371/journal.pone.0114781)
Supplement: Table S1 — Genes enriched in mutant group. This table includes 199 genes enriched in the mutant pri-miR-125a group. Embryo development related genes were taped using red letters. Cell proliferation regulation genes were marked with blue. (DOCX) [file pone.0114781.s001.docx]

Tab.S1 Genes enriched in mutant group

|  | log2 (Ratio) | | P-value(Differentially expressed) | |
| --- | --- | --- | --- | --- |
| Gene | Control/Normal | Mutation/Normal | Control/Normal | Mutation/Normal |
| ATP6V0E2 | 1.866079627 | 1.40125437 | 0.002496292 | 0.01898241 |
| ZNF689 | 1.34752416 | 1.729090913 | 0.01752721 | 0.003537411 |
| GSTZ1 | 2.924528677 | 1.996681775 | 0.000216816 | 0.019709835 |
| DNAJB9 | 3.239340129 | 1.619339797 | 1.66001E-05 | 0.007588813 |
| TAB1 | 2.528273122 | 3.476354865 | 0.000137514 | 8.05652E-06 |
| C3orf78 | 2.525845809 | 2.071646254 | 0.003370644 | 0.022115227 |
| POLR1A | 2.046844085 | 4.060466158 | 0.007158387 | 8.85299E-06 |
| C1orf115 | 1.576504364 | 3.03657979 | 0.049775016 | 0.000186111 |
| ORC1 | 1.81598444 | 1.236335446 | 0.00141433 | 0.019753883 |
| DEDD | 2.462415167 | 1.74585676 | 9.55022E-05 | 0.001530831 |
| CRYL1 | 1.813356157 | 3.461782297 | 0.046212096 | 5.43163E-05 |
| RAB36 | 1.002675655 | 1.411470502 | 0.029904528 | 0.0044049 |
| GALR2 | 1.976112499 | 2.492950097 | 0.023630163 | 0.003050806 |
| SLIT2 | 2.087586234 | 3.819041081 | 0.001204802 | 7.94614E-06 |
| SLC25A42 | 2.593422623 | 1.36483491 | 9.92785E-05 | 0.013773217 |
| ANKRD30BL | 2.559966761 | 2.771679185 | 7.01297E-05 | 3.92982E-05 |
| KAZALD1 | 2.235389837 | 3.781061477 | 0.001284392 | 1.04085E-05 |
| DDOST | 3.004696858 | 2.954699285 | 2.14038E-05 | 2.49984E-05 |
| RFWD3 | 1.069381555 | 1.343722911 | 0.041625254 | 0.013749577 |
| FOSB | 2.161434177 | 2.17918885 | 0.007481582 | 0.007305897 |
| ZNF385C | 1.589870315 | 2.325105887 | 0.003945682 | 0.000215758 |
| POM121\|POM121C | 1.632321623 | 1.528120216 | 0.015359989 | 0.024758332 |
| PPM1F | 1.513560607 | 1.727733211 | 0.014855438 | 0.00647788 |
| NPAS4 | 1.849138544 | 1.226618976 | 0.000567734 | 0.009621325 |
| ZNF317 | 2.656259657 | 3.006905574 | 0.000109466 | 0.000039825 |
| CCNA2 | 2.801403691 | 2.915915775 | 0.000124677 | 9.53868E-05 |
| PWP1 | 2.158688677 | 1.26464018 | 0.000296045 | 0.014314439 |
| TXNRD2 | 1.60679407 | 1.365688596 | 0.006999096 | 0.020688806 |
| STAMBP | 2.023112118 | 1.436752726 | 0.000317565 | 0.004006902 |
| ZNF524 | 1.177102452 | 1.820043882 | 0.03228388 | 0.002258402 |
| EIF3H | 1.670933861 | 1.383810959 | 0.001363381 | 0.005125954 |
| DCK | 1.91455258 | 1.733394494 | 0.000990538 | 0.002291955 |
| TFCP2 | 1.674239113 | 1.500315738 | 0.001390229 | 0.00309386 |
| RBBP8 | 2.485991106 | 1.909879724 | 0.000132771 | 0.00128972 |
| FUNDC2 | 1.751331922 | 1.363193668 | 0.0013804 | 0.008197259 |
| PROCR | 1.260570835 | 1.373208826 | 0.026978733 | 0.017634703 |
| ING3 | 1.878683464 | 3.116888946 | 0.001129159 | 2.06942E-05 |
| SART3 | 1.22387209 | 1.751907712 | 0.037495133 | 0.00440656 |
| ECE2 | 2.35054483 | 2.208995132 | 0.000257081 | 0.000471823 |
| MEGF8 | 2.285890532 | 2.443261607 | 0.000160658 | 9.86119E-05 |
| NPLOC4 | 3.262839641 | 1.730458119 | 1.47229E-05 | 0.002486444 |
| MAP4 | 1.446251594 | 2.669309795 | 0.047351301 | 0.000366791 |
| C7orf42 | 1.882888244 | 1.76723844 | 0.003420883 | 0.005786201 |
| TPBG | 2.286067348 | 1.967620179 | 0.001382964 | 0.005616406 |
| ASXL1 | 1.534902291 | 1.550433497 | 0.002387086 | 0.002279141 |
| DPM2 | 2.207805932 | 2.283027419 | 0.000226912 | 0.000183072 |
| PSMC4 | 1.870208966 | 2.742961518 | 0.003439407 | 0.000113975 |
| URGCP | 1.753996242 | 3.268097991 | 0.001651238 | 1.28949E-05 |
| NDEL1 | 1.820579081 | 1.166057782 | 0.000985623 | 0.019544564 |
| C1orf122 | 1.356225472 | 1.022377678 | 0.005980682 | 0.029197916 |
| CPD | 1.97211602 | 2.516845218 | 0.002695615 | 0.000337968 |
| BAZ1B | 3.165920465 | 1.536321077 | 6.28509E-05 | 0.005334082 |
| CYTH3 | 2.659808257 | 1.160180876 | 9.61811E-05 | 0.047656823 |
| PCNA | 2.818683358 | 3.459002342 | 3.49343E-05 | 8.69132E-06 |
| BSCL2\|HNRNPUL2-BSCL2 | 2.104223975 | 2.390679708 | 0.000347865 | 0.000130414 |
| ASNA1 | 1.784913516 | 1.300225853 | 0.001683583 | 0.015108656 |
| TPD52L2 | 1.763747659 | 1.52776084 | 0.000981824 | 0.002856655 |
| TSC2 | 1.420584647 | 1.547598498 | 0.0050369 | 0.002919515 |
| PTK7 | 1.635612816 | 1.259045226 | 0.003928579 | 0.022038532 |
| CSNK2A1 | 2.498545247 | 3.214397179 | 0.00040023 | 4.17325E-05 |
| TRUB2 | 3.118295358 | 3.059997327 | 9.16624E-05 | 0.000119407 |
| MED31 | 1.716410645 | 1.999960099 | 0.028101068 | 0.009737872 |
| FAM116B | 1.365125379 | 1.295248074 | 0.004828642 | 0.006777163 |
| NA | 2.260074985 | 1.969832856 | 0.000136599 | 0.000408331 |
| NDUFA10 | 4.56852988 | 3.90107862 | 2.30369E-06 | 5.28083E-06 |
| IP6K2 | 1.400805797 | 1.113271148 | 0.006119041 | 0.023753697 |
| SQLE | 3.53441114 | 2.378546917 | 4.64694E-05 | 0.007089134 |
| GSS | 1.169764486 | 1.060583814 | 0.012065562 | 0.020464728 |
| FHL1 | 3.208068431 | 3.131908214 | 4.31063E-05 | 5.90469E-05 |
| ZFAND2A | 1.239531998 | 2.110160878 | 0.01776018 | 0.000451284 |
| ITGA2B | 1.087235919 | 1.850873641 | 0.042621326 | 0.001647797 |
| WIPF2 | 3.998308465 | 3.375730474 | 5.45674E-06 | 1.91058E-05 |
| PLCXD1 | 1.843127987 | 1.190584431 | 0.000767915 | 0.015036925 |
| CCT7 | 2.185113475 | 1.640755928 | 0.005943074 | 0.049508046 |
| VANGL2 | 2.2227064 | 1.54125995 | 0.000962411 | 0.017764246 |
| H2AFY2 | 1.666768881 | 1.809793698 | 0.001409486 | 0.000774382 |
| SSU72 | 1.646197034 | 1.574308716 | 0.00166212 | 0.002358033 |
| C9orf9 | 1.14270786 | 2.060422888 | 0.034416355 | 0.000729476 |
| NA | 2.916476121 | 3.497669818 | 7.64319E-05 | 1.74186E-05 |
| PCMT1 | 3.046701469 | 2.693523701 | 0.000204356 | 0.000897121 |
| RNF2 | 3.410687389 | 2.7538465 | 1.81383E-05 | 0.000124844 |
| PTRF | 1.186677647 | 2.194923504 | 0.016460534 | 0.000225988 |
| ATP6V1F | 5.465431763 | 5.209794116 | 1.18511E-06 | 1.38565E-06 |
| POLR2J | 1.054127155 | 1.298704108 | 0.025509646 | 0.008321084 |
| TNKS1BP1 | 1.321269148 | 1.75453738 | 0.008710326 | 0.001280082 |
| RPIA | 2.234672274 | 1.264139333 | 0.000172203 | 0.010789308 |
| RAB43\|ISY1-RAB43 | 2.219084738 | 3.260807741 | 0.000599612 | 0.00002293 |
| H19 | 1.992991604 | 1.633321732 | 0.000456462 | 0.002180383 |
| UBE2N | 4.702011163 | 2.422278705 | 3.04063E-06 | 0.001489312 |
| TRMT1 | 1.633346285 | 2.915423737 | 0.009558846 | 7.89516E-05 |
| B3GAT3 | 1.970682877 | 1.266523876 | 0.001114844 | 0.025542999 |
| ABCC5 | 1.832368143 | 1.284592381 | 0.002783153 | 0.030935174 |
| EFHD1 | 1.017016468 | 1.645871714 | 0.028234432 | 0.001533793 |
| ZKSCAN3 | 2.557597665 | 1.401657406 | 0.000146767 | 0.016540142 |
| RUSC1-AS1 | 1.614494977 | 1.51106647 | 0.002271788 | 0.003769808 |
| SLC1A4 | 1.682816773 | 2.138751228 | 0.011976517 | 0.002690927 |
| ACP1 | 2.914856262 | 2.099083241 | 2.71073E-05 | 0.000376253 |
| AGFG2 | 1.86980583 | 1.162665904 | 0.000639451 | 0.015870856 |
| PRRC2C | 1.240764958 | 1.253768267 | 0.00982997 | 0.009368342 |
| PTPRF | 1.336735408 | 1.185510524 | 0.006548558 | 0.013614851 |
| NA | 3.808659026 | 3.662229166 | 6.00656E-06 | 7.93556E-06 |
| TAOK2 | 1.646952905 | 1.413751387 | 0.001610063 | 0.004753601 |
| TMED7 | 1.461351647 | 1.45733725 | 0.02172002 | 0.023601774 |
| MYB | 2.943538288 | 1.927682312 | 0.00011774 | 0.007531954 |
| CDT1 | 1.107073972 | 1.005184541 | 0.018206812 | 0.029671792 |
| DTWD1 | 1.858733146 | 1.306201069 | 0.001491965 | 0.01742724 |
| PCBD1 | 1.311163205 | 1.80279887 | 0.012523675 | 0.001497516 |
| MRPL18 | 1.943462902 | 1.560827531 | 0.000772006 | 0.004226889 |
| DGCR2 | 1.471832606 | 1.274015887 | 0.009080858 | 0.022239672 |
| BACH1 | 2.614618577 | 2.793737834 | 0.000130526 | 7.67203E-05 |
| POLR2E | 2.263380437 | 1.63856216 | 0.000135474 | 0.001651921 |
| LINC00294 | 1.713724544 | 2.563677325 | 0.009548169 | 0.00030312 |
| TBC1D23 | 2.299678228 | 1.897400825 | 0.000223138 | 0.00113331 |
| MRPL44 | 2.374925058 | 1.667374176 | 0.001071212 | 0.021106331 |
| ZNF395 | 1.577560412 | 1.533758432 | 0.005929403 | 0.007740027 |
| HOMER2 | 1.438520294 | 1.081969158 | 0.004953421 | 0.026445482 |
| MEGF8 | 1.455020125 | 4.64293994 | 0.027358163 | 2.97384E-06 |
| PIK3R3 | 1.339168657 | 2.017763282 | 0.025470136 | 0.001637994 |
| VPS4A | 1.860574568 | 2.688769464 | 0.00080517 | 4.63176E-05 |
| MTX1 | 1.293530305 | 1.70522668 | 0.008965814 | 0.00142553 |
| PRKAR1B | 2.838403399 | 3.656134657 | 6.13055E-05 | 9.31911E-06 |
| FAM64A | 1.372537793 | 1.137575145 | 0.013507153 | 0.038321994 |
| GPRIN1 | 4.645150816 | 3.249550318 | 3.64284E-06 | 0.000085717 |
| SLC22A17 | 1.384902448 | 2.402731517 | 0.005148363 | 8.66696E-05 |
| IL11RA | 1.832405169 | 1.952406865 | 0.00277353 | 0.00176645 |
| GALNS | 1.975479286 | 1.294167676 | 0.000540876 | 0.011298254 |
| NA | 3.166782566 | 2.605124563 | 1.32236E-05 | 5.56407E-05 |
| EEF2 | 1.957300098 | 2.024813749 | 0.000342359 | 0.000264615 |
| CHD8 | 1.330148068 | 1.578658632 | 0.007899311 | 0.002612278 |
| DAG1 | 3.383826013 | 2.227762587 | 1.28784E-05 | 0.000434339 |
| ACO2 | 1.381014573 | 2.251751167 | 0.008235642 | 0.000227552 |
| CWC25 | 3.879761276 | 3.553736556 | 0.000019649 | 0.000054993 |
| NA | 1.869203508 | 1.822075188 | 0.000884619 | 0.001137297 |
| SORBS3 | 2.014582177 | 1.509302171 | 0.002048753 | 0.017675305 |
| PABPN1\|BCL2L2-PABPN1 | 1.020203117 | 1.129293698 | 0.02823649 | 0.017203977 |
| MRPL27 | 2.957175899 | 1.651140259 | 2.59404E-05 | 0.002671168 |
| ASNS | 2.341479096 | 1.362907753 | 0.00012301 | 0.007088125 |
| SYMPK | 1.942681784 | 1.698171659 | 0.002481642 | 0.011045842 |
| JMY | 3.022488357 | 3.778106888 | 9.28792E-05 | 1.29172E-05 |
| IKBKAP | 1.286443802 | 1.607911201 | 0.010758407 | 0.002567167 |
| MORF4L1 | 2.949359558 | 2.549607279 | 5.68673E-05 | 0.000229431 |
| TLE3 | 1.039389348 | 1.018758295 | 0.026106753 | 0.029229756 |
| ATPAF1 | 3.330896005 | 1.63130775 | 2.46483E-05 | 0.01224261 |
| STIL | 1.485994228 | 1.406936645 | 0.004585435 | 0.006818855 |
| NUP107 | 1.614113328 | 1.731750083 | 0.003287202 | 0.002064789 |
| TRIM16\|TRIM16L | 1.634903671 | 1.645376016 | 0.0015254 | 0.001474074 |
| LDHB | 3.840078851 | 2.585844677 | 4.57427E-06 | 7.05859E-05 |
| SEMA6C | 1.385330924 | 1.959888186 | 0.005157951 | 0.000427256 |
| TRAFD1 | 1.505611705 | 1.442288278 | 0.002501825 | 0.003373802 |
| SLC35C2 | 1.230478638 | 2.11882546 | 0.012410683 | 0.000261996 |
| MFSD9 | 2.172472612 | 2.187178047 | 0.010659601 | 0.010628593 |
| CDC42BPA | 1.995014595 | 4.224252494 | 0.005895456 | 5.60225E-06 |
| NA | 1.720837184 | 1.430468924 | 0.001348248 | 0.005170635 |
| MFSD10 | 1.133211075 | 1.514939991 | 0.020799331 | 0.003711339 |
| SETD1A | 2.543323081 | 3.483323772 | 0.000176023 | 1.38426E-05 |
| SPEF1 | 1.316465411 | 1.44923618 | 0.013401479 | 0.007915916 |
| SNX15\|ARL2-SNX15 | 2.898181695 | 2.54091656 | 3.47766E-05 | 0.000107299 |
| ALG10B | 1.787555658 | 1.592121702 | 0.002733121 | 0.006746702 |
| UBA52 | 2.078408627 | 1.848522559 | 0.000717327 | 0.002012265 |
| CCDC75 | 1.410599094 | 1.725350252 | 0.04295814 | 0.013303329 |
| PTP4A3 | 1.755082283 | 2.655576948 | 0.001659072 | 6.31502E-05 |
| RPS23 | 3.685870265 | 2.585496194 | 4.60424E-06 | 4.59373E-05 |
| MIS18A | 1.595372899 | 1.29186273 | 0.005691134 | 0.022588806 |
| DNAJC8 | 3.430892751 | 1.550092339 | 1.71591E-05 | 0.015050033 |
| CCDC12 | 1.095048795 | 1.571388678 | 0.018074935 | 0.0019354 |
| SUMF2 | 2.040550974 | 2.213485827 | 0.002451869 | 0.001039409 |
| AP1G2 | 1.565229518 | 1.833646842 | 0.009769025 | 0.003362208 |
| TSPAN9 | 1.253318505 | 2.34897685 | 0.015606129 | 0.000165159 |
| NA | 1.711803327 | 1.604384878 | 0.006485648 | 0.015176447 |
| BCL2L13 | 1.304674274 | 1.533859016 | 0.030053433 | 0.0123982 |
| FURIN | 1.834924552 | 2.139702233 | 0.000578017 | 0.000178367 |
| GEMIN6 | 1.879464386 | 1.176235103 | 0.001105286 | 0.025585393 |
| NPC2 | 1.846667674 | 1.132595386 | 0.001535895 | 0.035839248 |
| KCNC4 | 3.055170625 | 1.765579576 | 2.52817E-05 | 0.002608266 |
| BAX | 1.375539312 | 1.765834799 | 0.005332846 | 0.00093713 |
| ARMC10 | 1.549684057 | 1.411378331 | 0.002954269 | 0.005772118 |
| PYCR2 | 1.300936384 | 1.344186532 | 0.008244046 | 0.006954566 |
| PTDSS1 | 1.440313006 | 1.718849043 | 0.004229559 | 0.001247716 |
| TIGD5 | 1.079899637 | 1.253451461 | 0.048435394 | 0.02432094 |
| PCIF1 | 3.478741604 | 2.779645414 | 1.03092E-05 | 5.81593E-05 |
| RAB40B | 3.058174914 | 2.002738666 | 0.000315304 | 0.02221008 |
| TRMT12 | 1.388191366 | 2.054102651 | 0.024310306 | 0.001511049 |
| MKRN1 | 2.75374849 | 1.694264476 | 9.51065E-05 | 0.006012976 |
| MINK1 | 1.701192975 | 1.98673057 | 0.002088628 | 0.000652712 |
| SDF2 | 1.799707261 | 1.411765426 | 0.001088462 | 0.006474137 |
| SLC41A3 | 1.510251196 | 1.593093 | 0.039225757 | 0.030266369 |
| CDK12 | 1.31614039 | 1.417663815 | 0.016948167 | 0.011986629 |
| MED22 | 1.391959645 | 1.427001014 | 0.013476961 | 0.012356086 |
| KLF2 | 2.030271089 | 2.149475432 | 0.000635514 | 0.000425319 |
| SDSL | 2.250940148 | 1.573088924 | 0.000167791 | 0.002708723 |
| PSMG3 | 3.490192456 | 3.714032941 | 6.08488E-06 | 4.37975E-06 |
| RAD9A | 2.208743868 | 1.869089855 | 0.000187371 | 0.000717866 |
| MYADM | 2.593798038 | 2.955851463 | 0.000223273 | 7.45819E-05 |
| EIF2B5 | 1.4908734 | 2.807133087 | 0.004478116 | 3.57343E-05 |
| AASS | 1.570318376 | 1.183701572 | 0.007281462 | 0.039160997 |
| GRN | 1.191075378 | 1.676102316 | 0.017196337 | 0.001978217 |
| ST20 | 1.226595217 | 2.360829074 | 0.044629775 | 0.000479561 |
| ABCF1 | 1.231634871 | 1.021615963 | 0.009131847 | 0.025092268 |
| NID1 | 1.736805875 | 2.138917351 | 0.009530311 | 0.001864867 |
| MEF2D | 1.378750218 | 4.106103305 | 0.015597072 | 4.23494E-06 |
| H2AFV | 1.523088183 | 2.049943019 | 0.008104133 | 0.000960021 |
| EEF1A2 | 1.306171567 | 2.670430291 | 0.014156264 | 0.000066512 |
| LPIN3 | 1.93949454 | 3.196630428 | 0.000768392 | 1.57955E-05 |
| CABIN1 | 1.919296716 | 3.910507481 | 0.006560888 | 1.01575E-05 |

Note. Embryo development related genes were taped using red letter. Cell proliferation regulation genes were marked with blue.
