# Supplementary material for: Functional Study of One Nucleotide Mutation in Pri-MiR-125a Coding Region which Related to Recurrent Pregnancy Loss
Source: PLoS One. 2014 Dec 5;9(12):e114781. doi: 10.1371/journal.pone.0114781 (PMC4257728; doi:10.1371/journal.pone.0114781)
Supplement: Table S2 — Genes diminished in mutant group. This table includes 141 genes diminished in mutant group. RNA processing associated genes are showed in red letters, and cell proliferation, migration and invasion genes used blue. (DOCX) [file pone.0114781.s002.docx]

Tab.S2 Genes diminished in mutant group

|  | log2 (Ratio) | | P-value(Differentially expressed) | |
| --- | --- | --- | --- | --- |
| Gene | Control/Normal | Mutation/Normal | Control/Normal | Mutation/Normal |
| RAPGEFL1 | -1.016779558 | -3.157383396 | 0.026033875 | 1.47164E-05 |
| LOC100507670 | -1.419609684 | -1.331818811 | 0.024612844 | 0.031160269 |
| UFD1L | -3.331904244 | -1.486636546 | 1.21903E-05 | 0.003118187 |
| UBE2M\|UBE2MP1 | -1.354313759 | -1.202722371 | 0.00667706 | 0.013239582 |
| SDF4 | -1.993813708 | -1.490570951 | 0.001253885 | 0.011518037 |
| CAPN1 | -2.828429626 | -1.452791809 | 3.23829E-05 | 0.003637353 |
| ALDH16A1 | -1.612272302 | -1.278278087 | 0.003607482 | 0.013478229 |
| HK1 | -1.744642018 | -1.127897581 | 0.00193779 | 0.022215636 |
| SP6 | -2.497504746 | -1.663377103 | 0.004184564 | 0.01112421 |
| ZNF207 | -3.131027501 | -1.352102368 | 5.77655E-05 | 0.008772628 |
| KLHL12 | -1.885396931 | -2.31877575 | 0.042456295 | 0.024946131 |
| AMBRA1 | -1.560696355 | -1.380512341 | 0.003516895 | 0.007426776 |
| CELSR2 | -3.125453933 | -2.086918769 | 0.000043581 | 0.000556252 |
| LINC00085 | -1.857363366 | -2.124153108 | 0.000955337 | 0.000380203 |
| SRRM3 | -2.361463473 | -2.135700805 | 0.001085034 | 0.004066243 |
| SMCR8 | -2.727361904 | -1.884536811 | 0.000176742 | 0.001571896 |
| XPO4 | -2.259803328 | -2.416733055 | 0.000276074 | 0.000177418 |
| IFITM2 | -3.267124214 | -2.502931395 | 2.03324E-05 | 0.000106881 |
| OR4K14 | -2.563276172 | -2.842810339 | 0.000194631 | 0.000155084 |
| PPP1R35 | -1.776021625 | -1.126548493 | 0.002073368 | 0.025853647 |
| LPCAT4 | -1.934050664 | -3.649863521 | 0.000439132 | 6.28846E-06 |
| LINC00263 | -1.061216432 | -1.356660482 | 0.045211434 | 0.020213597 |
| PRSS30P | -1.807097302 | -3.241687447 | 0.000949525 | 1.82153E-05 |
| VPS33B | -2.09151183 | -1.270365713 | 0.000390531 | 0.009677081 |
| C12orf75 | -1.065479918 | -1.607042364 | 0.044369251 | 0.018837702 |
| ATF7 | -2.024038766 | -2.636944133 | 0.001051882 | 0.000172971 |
| TJAP1 | -1.546699371 | -2.303222074 | 0.003051612 | 0.000199456 |
| DCST1 | -2.220004261 | -2.279772657 | 0.000765822 | 0.001001674 |
| GRINA | -3.395680014 | -1.873808448 | 1.28088E-05 | 0.000700633 |
| VGLL2 | -2.033605647 | -1.018337044 | 0.000327686 | 0.02635836 |
| SKIV2L2 | -1.767926697 | -2.890022611 | 0.00256262 | 0.000286997 |
| EIF1B | -1.116103179 | -1.285456561 | 0.017825635 | 0.008287328 |
| TMEM160 | -1.521365069 | -2.209283426 | 0.004294381 | 0.000396987 |
| PCGF1 | -2.105630981 | -1.945985252 | 0.009374309 | 0.009847109 |
| SON | -1.046647914 | -3.020149554 | 0.043564811 | 0.001158323 |
| MICB | -1.748769573 | -1.396899608 | 0.001856189 | 0.007486441 |
| LOC100499466 | -1.163685716 | -1.594844135 | 0.032490764 | 0.009230631 |
| PIK3R2 | -2.722874181 | -3.127350004 | 8.49218E-05 | 3.68185E-05 |
| SCAF1 | -2.474991086 | -1.045391491 | 9.22299E-05 | 0.02477316 |
| GNA11 | -1.583751945 | -2.557485397 | 0.003737951 | 0.000166865 |
| ZNF446 | -1.667976498 | -1.260007619 | 0.004141972 | 0.020860625 |
| SMYD2 | -1.954724879 | -2.108206594 | 0.000485371 | 0.00028205 |
| PCCB | -2.096753942 | -1.678054225 | 0.000446729 | 0.002057626 |
| MAP1A | -3.943406362 | -1.616851699 | 0.000200236 | 0.005307521 |
| MAF1 | -1.640555154 | -1.73495297 | 0.002713399 | 0.001929201 |
| SNRPB2 | -1.633942667 | -3.018114883 | 0.00274294 | 0.000295264 |
| ABHD11 | -1.68996743 | -1.171425626 | 0.001830009 | 0.016379964 |
| USP9X | -1.074532487 | -1.185622789 | 0.022707572 | 0.013926186 |
| NA | -4.561627113 | -4.513548183 | 4.58585E-06 | 6.53188E-06 |
| CA5B | -1.595961462 | -2.007945337 | 0.007751117 | 0.015448554 |
| QSOX1 | -1.313747732 | -1.522089063 | 0.006491898 | 0.002529429 |
| CPNE1 | -2.863482179 | -1.547432969 | 2.91301E-05 | 0.002361188 |
| USP36 | -1.494534068 | -1.177666067 | 0.0049965 | 0.018509291 |
| LOC541473 | -1.233483717 | -1.772906852 | 0.01205561 | 0.001301493 |
| SYDE1 | -3.378643506 | -1.502416542 | 7.37551E-05 | 0.005525412 |
| ALAS1 | -1.008093789 | -2.756388652 | 0.032646358 | 6.75216E-05 |
| AIP | -2.025766933 | -1.660184019 | 0.008107986 | 0.018606011 |
| PRAF2 | -1.901646986 | -1.125359884 | 0.00118248 | 0.024175361 |
| GTF3C2 | -1.384309331 | -1.869808803 | 0.010145034 | 0.001852252 |
| NCSTN | -2.467496501 | -2.182581621 | 9.47151E-05 | 0.000229722 |
| SNORA81 | -1.577304095 | -1.399573926 | 0.004718219 | 0.008940125 |
| TUBGCP2 | -1.140398262 | -2.056367006 | 0.024788581 | 0.001532799 |
| SNORA6 | -1.961932955 | -1.173858284 | 0.003123576 | 0.029241184 |
| SF3B14 | -1.032239283 | -1.366492685 | 0.035822053 | 0.009369981 |
| HLA-E | -1.321291223 | -1.610688778 | 0.017926563 | 0.006299486 |
| KLRG1 | -1.462123165 | -1.10148922 | 0.025649296 | 0.046838086 |
| NEIL1 | -1.71513828 | -1.572940919 | 0.001912653 | 0.00337639 |
| C11orf31 | -1.042676383 | -1.061600161 | 0.022396321 | 0.020523369 |
| AGPAT3 | -1.437309212 | -2.260019941 | 0.008535113 | 0.000791643 |
| SNAI1 | -2.230992006 | -2.355356683 | 0.00032956 | 0.000232212 |
| S1PR1 | -2.227272135 | -3.427358784 | 0.000573968 | 0.000507415 |
| U2AF1 | -1.433921789 | -1.558770089 | 0.007471911 | 0.004716257 |
| LMAN2 | -2.086072309 | -2.386519282 | 0.00023458 | 8.61414E-05 |
| C15orf27 | -1.672839831 | -1.215357693 | 0.001303329 | 0.010277034 |
| VAMP8 | -1.919928739 | -1.294035706 | 0.001763938 | 0.013917642 |
| ATP13A1 | -1.584035089 | -1.532654272 | 0.006300166 | 0.009749999 |
| WBP5 | -2.069170631 | -3.90245237 | 0.000672625 | 3.28018E-05 |
| COMP | -3.324409476 | -1.03662583 | 2.21899E-05 | 0.029689075 |
| OGFOD2 | -1.448548023 | -1.130130355 | 0.004849721 | 0.019544253 |
| SSU72 | -1.504583973 | -1.976503752 | 0.006815082 | 0.00139697 |
| OSBPL5 | -1.653465599 | -1.135340886 | 0.001268972 | 0.013980347 |
| CHCHD2 | -1.201932243 | -1.347182307 | 0.031847239 | 0.021175023 |
| HMGA2 | -4.24781596 | -2.47168603 | 4.5414E-06 | 9.32149E-05 |
| RNF185 | -2.046186519 | -1.838430974 | 0.000313955 | 0.000693427 |
| SNX12 | -2.946899182 | -2.114075186 | 5.21688E-05 | 0.000444161 |
| U2AF2 | -2.541393745 | -1.548628408 | 8.74886E-05 | 0.002822593 |
| SLIT1 | -1.955924378 | -1.636085251 | 0.000797073 | 0.002637605 |
| FOXJ2 | -1.456928869 | -1.20707585 | 0.015719727 | 0.036898553 |
| GNPDA1 | -1.771067212 | -2.42076161 | 0.002693805 | 0.000404817 |
| P4HA2 | -3.709228602 | -3.552048784 | 1.70691E-05 | 3.65138E-05 |
| SPIRE2 | -2.796169302 | -2.99092144 | 7.45658E-05 | 5.26249E-05 |
| KAT5 | -1.740694444 | -2.676801411 | 0.002806471 | 0.000407913 |
| CSRP2 | -1.612901455 | -1.995421093 | 0.005211018 | 0.001970168 |
| ALX4 | -1.252837655 | -1.560676849 | 0.035878971 | 0.025384707 |
| CAMKK2 | -1.947041399 | -1.585805129 | 0.002326032 | 0.004923968 |
| NA | -1.354570468 | -1.398469864 | 0.039916709 | 0.048364032 |
| CLPB | -1.296976436 | -1.154796695 | 0.007364003 | 0.014255275 |
| PDHA1 | -2.837335772 | -2.599441498 | 6.02057E-05 | 0.000136444 |
| PCCB | -1.251961298 | -1.313230647 | 0.00954711 | 0.007274275 |
| STK25 | -2.404076944 | -2.548775076 | 0.000248027 | 0.000210451 |
| CNOT4 | -1.774108922 | -1.750060547 | 0.001776643 | 0.001977782 |
| DHRS11 | -1.388887344 | -1.19064096 | 0.005161929 | 0.012741663 |
| SPHK2 | -2.2027044 | -1.392360207 | 0.000396272 | 0.007518419 |
| AKAP8 | -2.75939116 | -3.100012811 | 0.000226459 | 0.000185311 |
| BPNT1 | -3.397939224 | -2.577748361 | 5.68394E-05 | 0.000314326 |
| VHL | -1.838537302 | -2.368232974 | 0.002208896 | 0.00049582 |
| LOC100652996 | -2.311637767 | -3.406208201 | 0.000257791 | 2.67036E-05 |
| TNIP1 | -3.258522002 | -1.591030412 | 1.01506E-05 | 0.00169098 |
| NECAP2 | -1.641375378 | -1.255584246 | 0.002331603 | 0.011745878 |
| ZSCAN2 | -1.349690897 | -1.894237143 | 0.004967931 | 0.00045618 |
| PER1 | -6.021342744 | -6.005057855 | 1.14276E-06 | 1.1454E-06 |
| BAP1 | -1.787252715 | -1.636428884 | 0.000849698 | 0.001597399 |
| IBA57 | -1.952426467 | -1.177306591 | 0.000803169 | 0.017420605 |
| TIMP2 | -2.284914181 | -2.25490653 | 0.000191775 | 0.00021339 |
| STMN1 | -1.044607798 | -2.2249365 | 0.027398303 | 0.000261134 |
| PIGU | -2.340908056 | -1.184724652 | 0.000097743 | 0.011228707 |
| WBSCR22 | -1.198064167 | -1.002163324 | 0.020585271 | 0.045460373 |
| F8A1 | -2.383765466 | -1.931513857 | 0.000654166 | 0.003035408 |
| PYGO2 | -2.949486479 | -1.511832444 | 4.17198E-05 | 0.00367191 |
| ZDHHC3 | -1.621131917 | -1.212456364 | 0.001922802 | 0.011713413 |
| SLC39A3 | -2.963073992 | -1.109546526 | 2.17387E-05 | 0.016926192 |
| EDC3 | -2.880277133 | -2.297717211 | 0.000188002 | 0.000596097 |
| GUCA2B | -1.158960585 | -1.139800591 | 0.024266996 | 0.026821056 |
| IGSF8 | -3.134326023 | -2.1972433 | 2.02772E-05 | 0.00022268 |
| RAPH1 | -1.350381928 | -1.825914453 | 0.022637969 | 0.02754974 |
| TPPP | -2.358160991 | -3.303744747 | 0.00010126 | 1.07525E-05 |
| TAOK3 | -1.177367974 | -1.301031328 | 0.02113037 | 0.013185761 |
| TMEM180 | -2.901064411 | -1.896149451 | 4.47157E-05 | 0.000810672 |
| GNB2 | -1.576129448 | -2.145269662 | 0.003362574 | 0.000446632 |
| PCDHGC3 | -1.740519409 | -1.704928487 | 0.005646786 | 0.004178143 |
| CEP68 | -1.449714355 | -1.601827283 | 0.003717116 | 0.001919141 |
| NONO | -1.051091484 | -1.384885121 | 0.026947586 | 0.00636129 |
| STARD10 | -2.1164031 | -1.635455603 | 0.000282198 | 0.001770138 |
| ADAD2 | -2.668216426 | -3.317426314 | 0.000159692 | 0.0001369 |
| ASH2L | -1.254457019 | -1.426734997 | 0.008341212 | 0.00376341 |
| NADK | -2.942806869 | -3.871464594 | 2.32706E-05 | 5.0933E-06 |
| SEC61A2 | -1.207256723 | -1.614542298 | 0.010946618 | 0.001754499 |
| PTPN7 | -3.03692055 | -2.822986344 | 0.00062929 | 0.001194545 |
| SPSB2 | -2.388266257 | -1.676003911 | 0.000915843 | 0.006100921 |

Note. RNA processing associated genes are showed in red letter, and cell proliferation, migration and invasion genes used blue.
